# Total or Subtotal Hysterectomy for the Treatment of Endometriosis: A Review

**DOI:** 10.3390/jcm12113697

**Published:** 2023-05-26

**Authors:** Ibrahim Alkatout, Afrooz Mazidimoradi, Veronika Günther, Hamid Salehiniya, Leila Allahqoli

**Affiliations:** 1 Kiel School of Gynaecological Endoscopy, University Hospitals Schleswig-Holstein, Campus Kiel, Arnold-Heller-Str. 3, Haus 24, 24105 Kiel, Germany; veronika.guenther@uksh.de; 2 Student Research Committee, Shiraz University of Medical Sciences, Shiraz 7134814336, Iran; mazidimoradi.8836@gmail.com; 3 Social Determinants of Health Research Center, Birjand University of Medical Sciences, Birjand 9717853577, Iran; 4 Midwifery Department, Ministry of Health and Medical Education, Tehran 1467664961, Iran

**Keywords:** total hysterectomy, subtotal hysterectomy, supracervical hysterectomy, endometriosis, adenomyosis, review

## Abstract

Objective: The purpose of the review was to evaluate and compare outcomes after total or subtotal hysterectomy in women with endometriosis or adenomyosis. Methods: We searched four electronic databases: Medline (PubMed), Scopus, Embase, and Web of Science (WoS). The first aim of the study was to compare outcomes after total and subtotal hysterectomy in women with endometriosis, and the second aim was to compare the two procedures in women with adenomyosis. Publications that reported short- and long-term outcomes after total and subtotal hysterectomy were included in the review. The search was not subject to any limitation in terms of time or method. Results: After screening 4948 records, we included 35 studies published from 1988 to 2021; the studies were based on various methodologies. With regard to the first aim of the review, we found 32 eligible studies and divided these into the following four categories: postoperative short- and long-term outcomes, recurrence of endometriosis, quality of life and sexual function, and patient satisfaction after total or subtotal hysterectomy in women with endometriosis. Five investigations were deemed eligible for the second aim of the review. No differences were seen in terms of postoperative short- and long-term outcomes after subtotal or total hysterectomy in women with endometriosis or adenomyosis. Conclusions: Preservation or removal of the cervix in women with endometriosis or adenomyosis appears to have no effect on short- or long-term outcomes, recurrence of endometriosis, quality of life and sexual function, or patient satisfaction. Nevertheless, we lack randomized blinded controlled trials on these aspects. Such trials will be needed to enhance our comprehension of both surgical approaches.

## 1. Introduction

Endometriosis and adenomyosis are the most commonly encountered benign gynecological conditions. The exact prevalence of the two entities is unknown. Globally, endometriosis is estimated to occur in 10–15% of women of reproductive age [1] and approximately 50% of infertile women [2]. The prevalence of adenomyosis varies between 5% and 70% [2,3,4]. With no intervention, the rates of those who can conceive are almost 50%, 25%, and a few women with mild, moderate, and severe endometriosis, respectively [5]. The three types of endometriosis are ovarian endometrioma, superficial peritoneal endometriosis, and deep infiltrating endometriosis [6]. Typical symptoms of endometriosis include dysmenorrhea, dyspareunia, chronic pelvic pain, and reduced fertility [7]. Adenomyosis is marked by menorrhagia, pelvic pain, and dysmenorrhea [8]. Women with endometriosis or adenomyosis suffer from a variety of problems, including poor quality of life (QoL) due to severe pain or abnormal uterine bleeding [9,10,11]. The treatment of these benign conditions is determined individually [12], depending on symptoms [13], age, the desire for children [14,15], and the stage of the disease [15]. Medical therapy is usually the first option [16]. However, when medical treatments have failed and family planning has been completed, hysterectomy is offered to the patient for the purpose of relieving or alleviating pain [17,18]. Hysterectomy is associated with risks; the risk of complications is greater in women with endometriosis [19,20]. The majority of hysterectomies include removal of the cervix, but the rate of subtotal hysterectomies with retention of the cervical stump has increased in the last few decades [21,22]. Supporters of subtotal hysterectomy suggest that removal of the entire cervix might affect urinary function, reduce sexual satisfaction during intercourse, and interfere with pelvic floor support [23,24]. Nevertheless, recent publications have contradicted these assertions and revealed that, compared with total abdominal hysterectomy, subtotal hysterectomy did not improve outcomes in terms of urinary or sexual function. Not surprisingly, women who underwent subtotal hysterectomy were more likely to experience postoperative vaginal bleeding from the cervical stump until one year after surgery compared with women who underwent total abdominal hysterectomy [25,26,27]. The potential development of carcinoma on the cervical stump is a further cause of concern. The presence of cancer can be determined with relative accuracy only during the procedure [25].

Although subtotal hysterectomy is easier to perform and involves less extensive surgery, shorter operating times, and less perioperative bleeding [25], it does bear the risk of persistent pain and repeat surgery [22,28,29,30]. Several studies advise against subtotal hysterectomy in women with endometriosis, pelvic pain, or dysmenorrhea [22,28,29]. However, investigations that showed a risk of pelvic pain after subtotal hysterectomy did not include representative control groups [29,31,32]. Several randomized controlled clinical trials (RCTs) have demonstrated that outcomes after subtotal or total hysterectomy did not differ between the groups [33,34,35]. A randomized blinded controlled trial by Berner indicated that women with endometriosis and/or adenomyosis achieve symptomatic relief and improved QoL, regardless of whether the cervix is removed [32].

Based on these data, it appears that endometriosis would not be a contraindication to subtotal hysterectomy, unless retention of the cervix compromises the removal of the endometriosis [28,36]. However, the consensus arising from the few randomized trials that examined long-term outcomes of the two hysterectomy techniques is that subtotal hysterectomy provides no benefit over total hysterectomy in terms of sexual function, pain, or urinary symptoms [24,37,38]. In view of the above-mentioned controversial data, we performed a review to compare postoperative outcomes after total or subtotal hysterectomy in women with endometriosis or adenomyosis.

## 2. Materials and Methods

This systematic review was conducted according to the Preferred Reporting Items for Systematic Review and Meta-Analyses (PRISMA) guidelines [29], and the review was prospectively registered at PROSPERO (international prospective register of systematic reviews—PROSPERO registration ID: CRD42022353356.

### 2.1. Research Aims

The primary research aim was to evaluate and compare postoperative outcomes after total and subtotal hysterectomy in women with endometriosis.

The secondary aim was to evaluate and compare postoperative outcomes after total and subtotal hysterectomy in women with adenomyosis.

### 2.2. Information Sources and Search Strategy

We searched for relevant articles in the Medline (PubMed), Scopus, Embase, and Web of Science (WoS) databases. The following keywords were used for the search in September 2022: “hysterectomy”, “total hysterectomy”, “subtotal hysterectomy”, “partial hysterectomy”, “ supracervical hysterectomy”, “laparoscopic supracervical hysterectomy”, “total laparoscopic hysterectomy”, “laparoscopic hysterectomy”, “total abdominal hysterectomy”, “subtotal abdominal hysterectomy”, “abdominal hysterectomy”, “robotic supracervical hysterectomy”, “endometriosis and/or adenomyosis”, “benign disease”, and “benign indication”. MeSH keywords and Boolean (AND, OR) operators were employed to enhance the selection of entries.

### 2.3. Inclusion and Exclusion Criteria

We included all types of observational and clinical trials reporting outcomes of total and subtotal hysterectomy for endometriosis and adenomyosis, performed anywhere in the world and published in the English language. The search was not subject to any limitation in terms of time or method.

**Figure 1:**
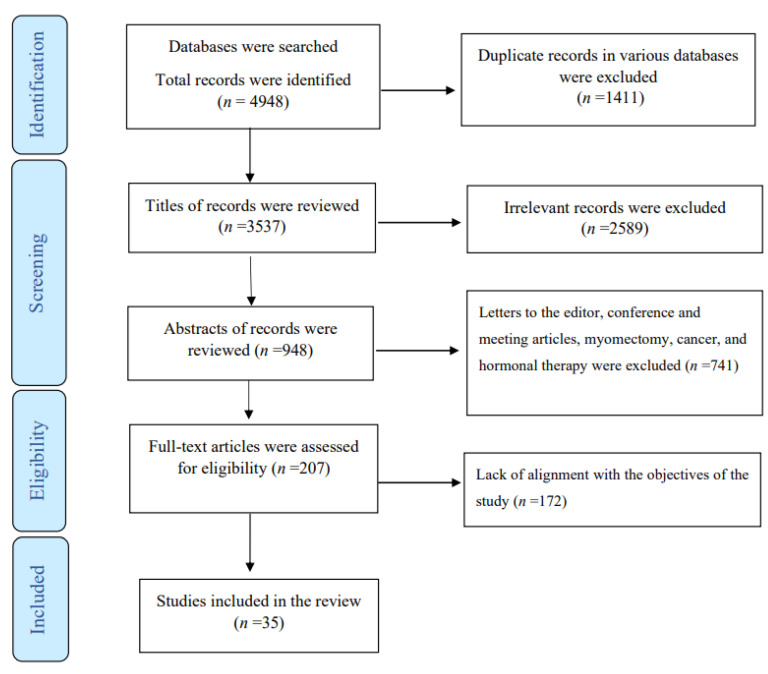
Flowchart of the study.

The following studies were excluded: experimental studies, those that focused on uterine artery coagulation, unembalmed cadavers, fibroids, myomectomy, adenocarcinoma, epithelial ovarian cancer, cervical and endometrial cancer, tumors, embolization, clear cell carcinoma, hormone therapy, intestinal endometriosis, local infiltration of bupivacaine and lidocaine, menopausal and postmenopausal women, medical treatment, extreme obesity, women with a catastrophic illness diagnosis for a mental disorder, endometrioid adenocarcinoma, transvaginal natural orifice transluminal endoscopic surgery, nerve-sparing radical hysterectomy, women with pelvic organ prolapse, morcellation of the uterus, or any additional surgery in conjunction with hysterectomy, as well as commentaries, letters to the editor, review, and rare case reports. Studies addressing endometriosis as a secondary process in scars after abdominal or pelvic surgical procedures were also excluded. The PRISMA flow chart illustrates the process of selection (Figure 1).

### 2.4. Study Selection

The EndNote software (EndNote X9, Thomson Reuters, Tokyo, Japan) was used to list the studies and screen them in accordance with the inclusion criteria. Study selection consisted of a screening phase, a selection phase, and a data abstraction phase. The first phase was conducted by three trained authors (LA, AMM, and IA) who screened titles/abstracts. Of the reviewed titles/abstracts, 207 articles were selected for a full-text review. Two authors (LA and IA) independently completed a checklist-style form and listed articles that met the inclusion criteria. The third author reviewed any discrepancies in the full-text review.

### 2.5. Data Extraction and Synthesis

Details of the articles were extracted using a standard form in order to ensure the consistency of this step for all investigations. Data such as the first author, year of publication, type of surgery, design of studies, indication for hysterectomy, and main results were independently extracted by the two writers. Any disagreement was clarified through discussion (with a third external collaborator if necessary). Due to the diverse modes of reporting, we performed a narrative synthesis of the studies.

## 3. Results

### 3.1. Study Selection

A total of 4948 publications, of which 1411 were duplicate articles, were found in the various databases. After reviewing the titles and abstracts, 4334 were excluded. Of the remaining articles, 579 were omitted due to lack of alignment with the objectives of the study. Finally, the review comprised 35 studies based on various methodologies: population-based studies, retrospective and prospective cohort studies, case–control studies, descriptive studies, randomized controlled trials, non-randomized interventional studies (quasi-experimental), cross-sectional surveys, case reports, and case series. Two reports provided no data about the study design.

### 3.2. Study Characteristics

Thirty-two studies were deemed eligible for the first aim of study, which was an evaluation and comparison of outcomes after total and subtotal hysterectomy in women with endometriosis. Five studies were considered eligible for the second aim of the study, which was an evaluation and comparison of outcomes after total and subtotal hysterectomy for adenomyosis. Two investigations [32,39] comprised women with endometriosis as well as those with adenomyosis and, thus, covered both aims of the review. The articles included in the review were published from 1988 to 2021. We contacted the authors of one study to clarify the reported results [40].

### 3.3. Synthesis of Results

#### 3.3.1. Evaluation and Comparison of Outcomes after Total and Subtotal Hysterectomy in Women with Endometriosis

Studies eligible for the first aim of study were divided into the following four categories: (a) postoperative short- and long-term outcomes (*n* = 17), (b) recurrence of endometriosis (*n* = 8), (c) QoL and sexual function (*n* = 8), and (d) patient satisfaction (*n* = 6). Some studies covered two or three outcomes [32,41,42,43,44,45]

##### Comparison of Postoperative Short- and Long-Term Outcomes of Total and Subtotal Hysterectomy in Women with Endometriosis

In 2014, Berner and co-workers reported that women with endometriosis had a more favorable outcome after total hysterectomy than those who underwent the subtotal procedure [46]. In line with these data [46], some studies concluded that preservation of the cervix during subtotal hysterectomy in patients with a preoperative diagnosis of endometriosis increases the risk of persistent postoperative pain [28,31]. However, a prospective study by Brucker et al. (2014) and another performed by Berner et al. (2015) reported no significant difference between subtotal and total hysterectomy in terms of reducing cyclic pelvic pain at 12 months after surgery or the occurrence and persistence of postoperative pain [32,42].

Vaginal bleeding after hysterectomy (total/subtotal) has been reported as a further undesirable outcome of surgery in women with endometriosis (0.92–25%) [46,47,48,49]. According to two studies, women who undergo subtotal hysterectomy are more likely to experience postoperative vaginal bleeding from the cervical stump [46,47]. Although Reznek and co-workers were unable to establish the actual cause of persistent vaginal bleeding in their patients, the authors concluded that subtotal hysterectomy is inadequate treatment for advanced endometriosis, and total hysterectomy should be considered in the majority of cases [50].

Nerve growth in women with endometriosis has been addressed in some investigations [51]. In a retrospective cohort study, Yunker et al. reported that the average nerve counts/high-power fields after subtotal hysterectomy in women with endometriosis were higher than those in women without the disease (17.7 vs. 14.6) [51]. Postoperative short- and long-term outcomes of total and subtotal hysterectomy in women with endometriosis are summarized in Table 1.

##### Comparison of Recurrent Endometriosis after Total or Subtotal Hysterectomy in Women with Endometriosis

The recurrence of endometriosis lesions or symptoms of the disease after surgery constitutes a serious challenge [46,59] and has been addressed in some studies [47,50,60,61,62,63,64]. The reported recurrence rates of endometriosis after hysterectomy (total/subtotal) range from 0% to more than 80% [47,50,60,61,62,63,64], partly due to varying definitions of recurrence, different stages of the disease, ovarian conservation, surgical procedure, and the length of the assessment period [47,50,60,61].

In a retrospective cohort study performed by Soliman et al., the estimated retreatment rates for endometriosis between 2004 and 2013 were 3.3%, 4.7%, and 5.4% in the 2nd, 5th, and 8th years, respectively, after total hysterectomy [61]. Matorras and co-workers reported a recurrence rate of 0 after total hysterectomy in 57 women with endometriosis [62]. Some studies reported lower or equivalent recurrence rates after subtotal hysterectomy compared with total hysterectomy in women with endometriosis [50]. The severity of endometriosis has been addressed in some investigations. In a retrospective study by Reznek et al., 55% of women with stage IV endometriosis who had undergone subtotal hysterectomy experienced recurrent symptoms similar to their preoperative complaints, thus contradicting the above-mentioned statement [50]. It was concluded that subtotal hysterectomy may be inadequate for advanced endometriosis, and total hysterectomy should be considered in cases of severe endometriosis [50,63]. Recurrence rates of endometriosis after total or subtotal hysterectomy are summarized in Table 2.

##### Comparison of QoL and Sexual Function after Total or Subtotal Hysterectomy in Women with Endometriosis

When considering the type of hysterectomy they wish to undergo, women are known to express concerns about sexual health and QoL [45]. Improved QoL and sexual function scores were reported in women with endometriosis who underwent hysterectomy, regardless of the type of surgery [32,45,66,67,68]. Although a study performed by Tan and co-workers revealed improvement of QoL after total abdominal hysterectomy in women with endometriosis [69], the majority of the investigations yielded no difference between the two surgical techniques with regard to QoL, bladder, or sexual function [32,45,66,68,69]. In a randomized controlled trial by Flory et al., sexual function did not differ between the subtotal and total hysterectomy groups [37]. A cross-sectional study by Pouwels et al. revealed that women’s sexual function after a mean follow-up period of 15.2 months was not related to retention or removal of the cervix at the time of hysterectomy [45]. Nevertheless, some authors did suggest the potential superiority of subtotal hysterectomy with regard to sexual function. In a retrospective investigation, Brucker et al. noted more rapid improvement of sexual activity and less dyspareunia (15.2% vs. 16.5%) in women with endometriosis after subtotal hysterectomy compared with those who underwent total hysterectomy [42]. The comparison of QoL and sexual function after total or subtotal hysterectomy in women with endometriosis is summarized in Table 3.

##### Comparison of Patient Satisfaction after Total or Subtotal Hysterectomy in Women with Endometriosis

Although the majority of women who underwent hysterectomy were satisfied with the outcome regardless of cervix removal or retention [32,44,70], we have scarce data about patient satisfaction or regret associated with total or subtotal hysterectomy in women with endometriosis [43,71].

In a cross-sectional study, Pouwels and co-workers reported no difference in patient satisfaction between the two treatment groups at 12 months after the procedures [45].

Critics of subtotal hysterectomy appear to be concerned about the risk of cervical stump symptoms, such as postoperative pelvic pain or vaginal bleeding, causing distress and eventually necessitating repeat surgery, whereas critics of total hysterectomy are concerned about the risk of pelvic organ prolapse.

Nevertheless, studies demonstrated that, although cervical stump symptoms (frequent occurrence of vaginal bleeding and pelvic pain) are relatively common after subtotal hysterectomy, the overall level of patient satisfaction is high [31,43]. A comparison of patient satisfaction after total or subtotal hysterectomy in women with endometriosis is summarized in Table 4.

#### 3.3.2. Evaluation and Comparison of Outcomes after Total or Subtotal Hysterectomy in Women with Adenomyosis

Five studies were eligible for the second aim of the study [32,39,44,73,74]. In women with adenomyosis, studies addressing long-term outcomes after hysterectomy (total or subtotal) reported no benefit of subtotal hysterectomy over total hysterectomy in terms of pelvic pain reduction, patient satisfaction, or QoL at 12 months post-surgery [32,73,74].

In 2010, Ajao et al. registered no difference in the persistence of symptoms or overall satisfaction in women with adenomyosis on the basis of whether the cervix was removed or retained at the time of surgery. However, a higher percentage of patients who underwent subtotal hysterectomy reported an improvement in their postoperative QoL (92.1% vs. 76.5%) as well as the incidence of persistent bleeding (13.2% vs. 8.5%). After adjusting for potential confounders, no differences were seen between subtotal and total hysterectomy with regard to the persistence of symptoms (abnormal bleeding, dyspareunia, pelvic pain, or pelvic fullness/pressure) [73]. A comparison of outcomes after total hysterectomy in women with adenomyosis is summarized in Table 5.

## 4. Discussion

The optimal surgical procedure for endometriosis or adenomyosis is controversially discussed. One of the main issues is removal of the cervix. The purpose of the present review was to evaluate and compare outcomes after total or subtotal hysterectomy in women with endometriosis or adenomyosis. The body of published data comparing the two approaches in women with endometriosis or adenomyosis is limited. After a comprehensive search, we found 35 published studies from 1988 to 2021. The eligible articles reported contradictory data with regard to surgical outcomes after total and subtotal hysterectomy [29,31,32,41,42,43,44,48,51,53,55,56,57,58,59].

Some authors state that preserving the cervix during subtotal hysterectomy in women with endometriosis increases the risk of persistent postoperative pain [28,31] or vaginal bleeding from the cervical stump [46,47], whereas others report no significant difference between the two procedures in terms of reducing cyclic pelvic pain at 12 months after surgery nor in the occurrence and persistence of postoperative pain [32,42].

Okaro and co-workers (2001) stated that subtotal hysterectomy “is not necessarily the right procedure for women with pelvic pain” and that this procedure should be viewed with extreme caution in women with endometriosis [29]. Berner et al. (2014) reported a major reduction in cyclic pelvic pain after subtotal hysterectomy in women with endometriosis as well as those with adenomyosis. The results of the latter study support the view of gynecologists who consider subtotal hysterectomy an adequate procedure in women with benign disorders [28,36].

Regardless of the type of surgery, persistent bleeding after hysterectomy has been reported in 0.92% to 25% of the cases [46,47]. The existing body of data is inconclusive with regard to risk factors for persistent postoperative cervical stump bleeding [48]. Although endometriosis was suggested as a risk factor for postoperative bleeding in 1999 [49], a recent report failed to show an association between postoperative bleeding and endometriosis [39]. Some authors have claimed that the higher incidence of bleeding after subtotal hysterectomy can be accounted for only by an inappropriate surgical technique [50]. However, postoperative bleeding appears to be related to the indication for surgery as well. When hypermenorrhea is the indication of surgery, postoperative spotting would be common. It should be noted that spotting after a hysterectomy does not require treatment. Preservation of the cervix bears the risk of cervical stump carcinoma. As cervical stump symptoms appear to be rather common after subtotal hysterectomy, women should be informed preoperatively of the risk of persistent menstrual bleeding and/or pain.

One of the outcomes addressed in the present review is the recurrence of endometriosis after total or subtotal hysterectomy. Endometriosis may recur after either surgical technique [47,50,60,61,62,63,64]. Some authors suggest that subtotal hysterectomy may be inadequate for the treatment of endometriosis, especially advanced endometriosis, and total hysterectomy should be considered in most cases [50,63]. Yet, some women who have undergone total hysterectomy need to undergo repeat treatment [47,50,60,61,62,63,64]. Although the exact incidence of persistent endometriosis after surgery is not known [55], some authors have suggested that preservation of the ovaries or aggressive excision of all endometriotic implants at the time of hysterectomy is the main factor in persistent endometriosis [75,76]. Namnoum et al. concluded that recurrent pain and the risk of reoperation due to endometriosis were higher in women who had ovarian preservation compared with those who did not [57]. However, in a study performed by Sandström and co-workers, the proportion of women with pelvic or lower abdominal pain was significantly reduced, but the reduction was seen independent of whether bilateral oophorectomy was performed [40]. Further research will be needed to evaluate the origins of recurrence and the risk factors associated with retreatment. Some authors have observed higher rates of retreatment in younger patients than in older ones [61,77]. It should be noted that all reported recurrences were clinically relevant; yet, other recurrences may have remained undetected due to asymptomatic disease [62]. On the other hand, the recurrence of pain does not necessarily imply recurrence of endometriosis [78].

Another important outcome evaluated in the present study was QoL and sexual function after total or subtotal hysterectomy in women with endometriosis. Contradictory data have been reported in this regard [32,45,66,68,69]. Some authors observed no inter-group differences in QoL or sexual function after subtotal or total hysterectomy [37]. Berner et al. registered no difference in total QoL scores in the long term between total and subtotal hysterectomy in women with or without endometriosis or in those with or without adenomyosis [32]. Although the above-mentioned reports mentioned no difference between the two procedures with regard to QoL or sexual function, either in the short or the long term [79,80], some authors did suggest a possible advantage for subtotal hysterectomy. In a retrospective study by Brucker et al. [42] and a randomized study by Ellström Engh et al. [81], women with endometriosis in the subtotal hysterectomy group experienced a more rapid improvement of their sexual activity and had less dyspareunia compared with women who underwent total hysterectomy. According to Lermann et al., women undergoing subtotal hysterectomy had the highest sexual function scores based on the Brief Profile of Female Sexual Function (B-PFSF) questionnaire compared with those who underwent total hysterectomy, but the difference was not statistically significant [42].

Differences in the reported outcomes of sexual function have been attributed to vaginal vault pain, vaginal shortening, changes in cervicovaginal innervation, and the absence of cervical mucus production in women who underwent total hysterectomy [82,83]. The interpretation of published data on this topic is limited by difficulties in the standardization of studies and the heterogeneity of patients. Any potential difference in QoL and sexual function after total or subtotal hysterectomy in women with endometriosis must be addressed in trials specifically designed for this purpose.

Patient satisfaction or regret after total or subtotal hysterectomy in women with endometriosis was investigated because of the paucity of published data on the subject. Although some prospective observational trials confirmed a high degree of patient satisfaction after subtotal hysterectomy [31,36,49], critics of subtotal hysterectomy expressed concerns about the risk of cervical stump symptoms, such as vaginal bleeding and pelvic pain, causing patient distress and eventually necessitating repeat surgery [31]. However, these concerns were not confirmed in other studies. Despite the prevalence of cervical stump symptoms after either surgical procedure, the degree of overall patient satisfaction after subtotal hysterectomy was reported to be high [43]. The frequent occurrence of vaginal bleeding and pelvic pain after subtotal hysterectomy also did not affect patient satisfaction [31,84]. However, the validity of this conclusion is limited by the absence of a reference group in some studies.

Due to the poor results of endometrial ablation or hormone treatment, uterine adenomyosis usually requires hysterectomy [85]. Since adenomyosis is related primarily to the uterine corpus, both total and subtotal hysterectomy are potential treatment options for this condition. However, the existing body of data on the long-term outcomes of these interventions is scarce. Compared with total hysterectomy, cervical retention was believed to be associated with fewer adverse surgical outcomes related to sexual or urinary function, and these factors did play a role in the patient’s decision to undergo subtotal hysterectomy [73]. The existing data suggest no difference in these outcomes and reveal no potential advantage in favor of cervical retention [80,86]. In a prospective study, Ajao et al. registered no apparent difference between those who underwent cervical removal or retention [73]. However, a higher percentage of patients who underwent subtotal hysterectomy were reported to have experienced an improvement in their postoperative QoL [34,87]. In a retrospective cohort study and follow-up survey comprising 249 patients, Ajao et al. noted that retention of the cervix did not appear to increase the risk of persistent symptoms [74]. Berner (2015) also registered no difference between the two allocated treatment groups with regard to pelvic pain reduction, patient satisfaction, or QoL at 12 months after hysterectomy in women with adenomyosis [32]. In a randomized clinical trial with a 14-year questionnaire follow-up, Andersen et al. confirmed that subtotal abdominal hysterectomy was not superior to total abdominal hysterectomy with regard to any outcome [79].

## 5. Limitations and Recommendations

In the present review, we compared outcomes after total or subtotal hysterectomy in women with endometriosis or adenomyosis. The investigations differed in terms of the stage of endometriosis, the age of patients, sample size, ovarian preservation, follow-up periods, and the quality of studies. Since few randomized controlled trials have been performed on the subject, our review does not provide a comparative analysis as to which surgical procedure is preferable, but it does present the data of each procedure on the basis of the measured outcomes. It should be noted that the criteria for recommending subtotal or total hysterectomy remain ambiguous. We conclude that there is an urgent need for further prospective randomized trials on subtotal versus total hysterectomy for endometriosis and adenomyosis in order to better evaluate clinical outcomes, complications, postoperative QoL, sexual function, and patient satisfaction. In designed trials, the factors of age, ovarian preservation, and elective and emergency surgery should be controlled. Some studies have shown that women who undergo hysterectomy for endometriosis before the age of 30 years are more likely to experience residual symptoms than older women. Furthermore, some authors have mentioned that concomitant bilateral salpingo-oopherectomy during hysterectomy could be one of the main factors affecting patient outcomes.

## 6. Conclusions

The present review yielded contradictory data. To our knowledge, the discrepancies have not been addressed in randomized blinded controlled trials. The review revealed that cyclic pain, patient satisfaction, and QoL did not differ after subtotal or total hysterectomy in women with endometriosis or adenomyosis. RCTs with long-term follow-up periods and high-quality outcome data will be needed to compare subtotal versus total hysterectomy and shed light on clinical outcomes, complications, postoperative QoL, and sexual function. Pending the availability of robust data concerning essential outcomes after subtotal and total hysterectomy, we recommend that preference be given to the safer surgical method in the respective setting.

## Figures and Tables

**Table 1:** Characteristics of studies that reviewed outcomes in women with endometriosis after total or subtotal hysterectomy.

First Author/Year	Sample Size (*n*)	Type of Surgery	Design	Indication	Main Results
Brunes, M., et al., 2021 [52]	1074 cases of endometriosis, 10,890 cases without endometriosis	Total hysterectomy	Nationwide cohort study	Benign uterine disease	The long-term prescription of analgesics was not reduced after hysterectomy in women with endometriosis.
Sandström, A., et al., 2020 [40]	137	Subtotal (*n* = 6) and total hysterectomy (*n* = 131)	Population-based registry study	Endometriosis	Pelvic or lower abdominal pain was reduced by both methods.
Inés Poveda, G., et al., 2016 [53]	22	Total hysterectomy	Retrospective study	Deep endometriosis	All of the variables, especially chronic pelvic pain, improved significantly after surgery.
Yunker, A., et al., 2015 [51]	35 (*n* = 8 cases of endometriosis)	Subtotal hysterectomy	Retrospective cohort study	Endometriosis and benign indications	Average nerve counts/high-power fields were 17.7 vs. 14.6 in patients with and without endometriosis, respectively.
Berner, E., et al., 2015 [32]	62 (*n* = 15 cases of endometriosis)	Total (*n* = 7) and subtotal hysterectomy (*n* = 8)	Randomized blinded controlled trial	Dysmenorrhea	Reduction of cyclic pelvic pain 12 months after hysterectomy did not differ between groups.
Berner et al., 2014 [44]	105	Subtotal hysterectomy (*n* = 14)	Prospective observational study	Endometriosis	Cyclic pelvic pain was reduced to a minimum by 12 months and was associated with high patient satisfaction.
Brucker, Sara Y., et al., 2014 [42]	915 (*n* = 84 cases of endometriosis)	Subtotal (*n* = 67) and total hysterectomy (*n* = 17)	Prospective, questionnaire-based follow-up study	Benign uterine disease	There was no significant difference in the occurrence and persistence of postoperative pain.
Lieng, M., et al., 2010 [43]	449 (*n* = 64 cases of endometriosis)	Subtotal hysterectomy	Retrospective study	Benign condition	The mean pain score was significantly reduced.
Lieng, M., et al., 2008 [31]	240	Subtotal vs. total hysterectomy	Retrospective study	Benign conditions	The data revealed a high level of patient satisfaction with subtotal hysterectomy.
Ghomi, A., et al., 2005 [39]	64 (*n* = 22 cases of endometriosis)	Subtotal hysterectomy	Prospective	Benign conditions	23% of women with endometriosis reported cyclic bleeding at 15 months after surgery.
Fedele, L., et al., 2005 [54]	38	Total hysterectomy (*n* = 26)	Descriptive study	Symptomatic recurrences of deep endometriosis	31% of patients reported recurrent pain.
Ford, J., et al., 2004 [41]	60	Total hysterectomy (*n* = 9)	Cohort study	Rectovaginal endometriosis	Patients achieved a considerably higher cure rate.
Okaro, E. O., et al., 2001 [29]	70 (*n* = 5 cases of endometriosis)	Subtotal hysterectomy	Retrospective study	Menorrhagia and dysmenorrhea	Symptoms related to the cervical stump were seen in all women with endometriosis.
Kim, K. S., et al., 2001 [55]	1	Total hysterectomy	Case report	Endometriosis	Persistent endometriosis with vaginal and sigmoid–colonic invasion after total hysterectomy.
Nezhat, C. H., et al., 1996 [56]	6	Subtotal hysterectomy	NA	Persistent pelvic pain and endometriosis	Five women had extensive pelvic endometriosis despite subtotal hysterectomy.
Namnoum, A., B. et al., 1995 [57]	138	Total hysterectomy	Historical prospective study	Endometriosis	21% of women had recurrent pain and needed repeat surgery.
Reich, H., et al., 1994 [58]	52	Total hysterectomy	NA	Endometriosis	The majority of women achieved significant to complete relief from pelvic pain postoperatively.

Abbreviations: *n*: number; N/A: not available.

**Table 2:** Characteristics of studies that addressed recurrence rates of endometriosis after total or subtotal hysterectomy.

First Author/Year	Sample Size (*n*)	Type of Surgery	Design	Indication	Main Results
Balasubramaniam, et al., 2020 [60]	70	Total hysterectomy	Retrospective study	Stage IV endometriosis	The recurrence rate during the follow-up period was 0.
Shirane, A., et al., 2019 [47]	807	Total hysterectomy	Retrospective cohort	Endometriosis	The recurrence rate of endometriosis was 2.47%.
Soliman, A. M., et al., 2017 [61]	24,915	Total hysterectomy	Retrospective cohort	Endometriosis	Estimated retreatment rates were 3.3%, 4.7%, and 5.4% in the 2nd, 5th, and 8th years after hysterectomy, respectively.
Schuster, M. W., et al., 2012 [65]	464 (*n* = 102 cases of endometriosis)	Subtotal hysterectomy	Case–control Study	Benign indications	Repeat surgery rates were 2.9% and 3.3% in women with and without endometriosis, respectively.
Matorras, R., et al., 2002 [62]	172	Total hysterectomy (*n* = 57)	Prospective randomized trial	Endometriosis	The recurrence rate was 0.
Reznek, M. A., et al., 1999 [50]	9	Subtotal hysterectomy	Retrospective	Stage IV endometriosis	Recurrent symptoms were reported in 55.5% of cases.
Rana, N., et al., 1996 [63]	1	Subtotal hysterectomy	Case reports	Severe pelvic endometriosis	Recurrence of symptomatic endometriosis was reported.
Dmowski, W. P., et al., 1988 [64]	7	Total hysterectomy	Case series	Endometriosis	Recurrences were reported in 85.7% of cases.

**Table 3:** Characteristics of studies that addressed quality of life/sexual function in women with endometriosis after total or subtotal hysterectomy.

First Author/Year	Sample Size (*n*)	Type of Surgery	Design	Indication	Main Results
Ala-Nissilä et al., 2017 [66]	212 (*n* = 21 cases of endometriosis)	Subtotal (*n* = 13/107) and total hysterectomy (*n* = 8/105)	Non-randomized cohort study	Benign indications	The rates of subjective urinary and sexual symptoms or subsequent operations for urinary incontinence and genital prolapse did not differ between groups.
De la Hera-Lazaro, Cristina M., et al., 2016 [67]	46	Total hysterectomy	Non-randomized interventional study (quasi experimental)	Stage IV endometriosis	The patients’ QoL was improved.
Pouwels, N. S. A., et al., 2015 [45]	212 (*n* = 57 cases of endometriosis)	Total (*n* = 29/61) and subtotal hysterectomy (*n* = 28/54)	Cross-sectional survey	Benign conditions	Satisfaction with sexual function did not differ between groups.
Berner, E., et al., 2015 [32]	62 (*n* = 15 cases of endometriosis)	Total (*n* = 7/31) and subtotal hysterectomy (*n* = 8/28)	Randomized blinded controlled trial	Dysmenorrhea	QoL did not differ between the two groups at 12 months after hysterectomy.
Radosa, J. C., et al., 2014 [68]	237 (*n* = 170 cases of endometriosis)	Total hysterectomy (*n* = 98) and subtotal hysterectomy (*n* = 72)	Observational cohort study	Benign uterine disease	Postoperative sexual function did not differ between groups.
Brucker, Sara Y., et al., 2014 [42]	915 (*n* = 84 cases of endometriosis)	Subtotal hysterectomy (*n* = 67/788) and total hysterectomy (*n* = 17/127)	Prospective, questionnaire-based follow-up study	Benign uterine disease	QoL and sexual function parameters were better in the subtotal hysterectomy group than in the total hysterectomy group.
Tan, B. K., et al., 2013 [69]	16	Total hysterectomy	Retrospective	Endometriosis	Total hysterectomy significantly improved HRQoL.
Ford, J., et al., 2004 [41]	60 (*n* = 10 cases underwent hysterectomy)	Total hysterectomy (*n* = 9/10)	Cohort study	Rectovaginal endometriosis	Patients who underwent total hysterectomy reported a favorable response and had normal QoL.

Abbreviations: HRQoL, health-related quality of life, *n*: number, QoL: quality of life.

**Table 4:** Characteristics of studies that addressed satisfaction in women with endometriosis who underwent total or subtotal hysterectomy.

First Author/Year	Sample Size	Type of Surgery	Design	Indication	Main Results
Srichaikul, P., et al., 2018 [72]	1092 (*n* = 486 cases of endometriosis)	Total hysterectomy	Retrospective descriptive study	Benign indication	94.4% of the patients were extremely satisfied with the outcome of surgery.
Pouwels N.S.A, et al., 2015 [45]	212 (*n* = 57 cases of endometriosis)	Total (*n* = 29/61) and subtotal hysterectomy (*n* = 28/54)	Cross-sectional survey	Benign conditions	Satisfaction did not differ between groups.
Berner, E., et al., 2015 [32]	62 (*n* = 15 cases of endometriosis)	Total (*n* = 7/31) and subtotal hysterectomy (*n* = 8/31)	Randomized controlled trial	Dysmenorrhea	Patient satisfaction at 12 months after hysterectomy did not differ between groups.
Schiff et al., 2015 [70]	228 (*n* = 12 cases of endometriosis)	Total (*n* = 11/156) and subtotal (*n* = 3/72) hysterectomy	Prospective cohort	Benign indication	Cervix removal was not associated with satisfaction or well-being in the course of recovery.
Berner, E., et al., 2014 [44]	105	Subtotal hysterectomy (*n* = 14/105)	Prospective observational study	Women with perioperatively detected endometriosis and women with histologically proven adenomyosis	A high level of patient satisfaction was reported after subtotal hysterectomy.
Lieng, M., et al., 2010 [43]	449 (*n* = 64 cases of endometriosis)	Subtotal hysterectomy (*n* = 64)	Retrospective study	Benign conditions	Women were generally satisfied with the outcome of the subtotal procedure.

**Table 5:** Characteristics of studies that addressed outcomes after total or subtotal hysterectomy in women with adenomyosis.

First Author/Year	Sample Size	Type of Surgery	Design	Indication	Main Result
Ajao, M. O., et al., 2019 [73]	443 (*n* = 171 cases of adenomyosis)	Total or subtotal hysterectomy	Retrospective cohort study and follow-up survey	Benign indications	No difference was reported in the persistence of symptoms with or without cervix removal. Retention of the cervix did not increase the risk of persistent symptoms or patient satisfaction.
Ajao, M. O., et al., 2016 [74]	623 (*n* = 249 cases of adenomyosis)	Total or subtotal hysterectomy	Retrospective cohort study and follow-up survey	Adenomyosis	Persistent bleeding and post-procedural pain were noted in 10.7% and 16.2% of patients, respectively. Outcomes and the persistence of symptoms did not differ between groups.
Berner, E., et al., 2015 [32]	62 (*n* = 27 cases of adenomyosis)	Total (*n* = 15) or subtotal hysterectomy (*n* = 12)	Randomized blinded controlled trial	Dysmenorrhea	Pelvic pain reduction, patient satisfaction, and QoL at 12 months did not differ between groups.
Berner, E., et al., 2014 [44]	113 (*n* = 19 cases of adenomyosis)	Subtotal hysterectomy	Prospective observational study	Endometriosis and women with histologic confirmation of adenomyosis	Subtotal hysterectomy was associated with high patient satisfaction and reduced cyclic pelvic pain to a minimum within 12 months after the procedure.
Ghomi, A., et al., 2005 [39]	64 (*n* = 20 cases of adenomyosis)	Subtotal hysterectomy	Prospective	Uterine leiomyomata, abnormal uterine bleeding, dysmenorrhea, or chronic pelvic pain	20% of women with adenomyosis reported cyclic bleeding at 15 months after surgery.

Abbreviations: *n*: number, QoL: Quality of life.

## Data Availability

The datasets analyzed for the current study are available from the corresponding author on reasonable request.
